# Hepatitis C Virus Induces MDSCs-Like Monocytes through TLR2/PI3K/AKT/STAT3 Signaling

**DOI:** 10.1371/journal.pone.0170516

**Published:** 2017-01-23

**Authors:** Naicui Zhai, Haijun Li, Hongxiao Song, Yang Yang, An Cui, Tianyang Li, Junqi Niu, Ian Nicholas Crispe, Lishan Su, Zhengkun Tu

**Affiliations:** 1 Institute of Translational Medicine, the First Hospital of Jilin University, Changchun, China; 2 Department of Hepatobiliary and Pancreatic Diseases, the First Hospital of Jilin University, Changchun, China; 3 Department of Pathology, University of Washington, Seattle, Washington, United States of America; 4 Lineberger Comprehensive Cancer Center, School of Medicine, University of North Carolina at Chapel Hill, Chapel Hill, North Carolina, United States of America; Korea Advanced Institute of Science and Technology, REPUBLIC OF KOREA

## Abstract

**Background and Aims:**

Recent studies reveal the accumulation of myeloid derived suppressor cells (MDSCs) in human peripheral blood mononuclear cells (PBMCs) following HCV infection, which may facilitate and maintain HCV persistent infection. The mechanisms by which HCV induces MDSCs are poorly understood. In the present study, we investigated the mechanisms by which HCV induces MDSCs that lead to suppression of T cell proliferation and expansion of CD4^+^Foxp3^+^ regulatory T cells.

**Methods:**

Purified monocytes from healthy donors were cultured with HCV core protein (HCVc) or cell culture-derived HCV virions (HCVcc), and characterized the phenotype and function of these monocytes by flow cytometry, quantitative PCR, ELISA and western blot assays. In addition, peripheral blood from healthy donors and chronic HCV infected patients was collected, and MDSCs and CD4^+^CD25^+^CD127^-^ regulatory T cells were analyzed by flow cytometry.

**Results:**

Both HCVc and HCVcc induced expression of IDO1, PD-L1 and IL-10, and significantly down-regulated HLA-DR expression in human monocytes. HCVc-treated monocytes triggered CD4^+^Foxp3^+^ Tregs expansion, and inhibited autologous CD4^+^ T cell activation in an IDO1-dependent fashion. Our results showed that HCV virions or HCV core proteins induced MDSC-like suppressive monocytes via the TLR2/PI3K/AKT/STAT3 signaling pathway. Monocytes derived from patients with chronic HCV infection displayed MDSCs characteristics. Moreover, the percentages of CD14^+^ MDSCs and CD4^+^CD25^+^CD127^-^ Tregs in chronic HCV infected patients were significantly higher than healthy individuals, and the frequency of MDSCs correlated with CD4^+^CD25^+^CD127^-^ Tregs.

**Conclusions:**

HCV induced MDSC-like suppressive monocytes through TLR2/PI3K/AKT/STAT3 signaling pathway to induce CD4^+^Foxp3^+^ regulatory T cells and inhibit autologous CD4^+^ T cell activation. It will be of interest to test whether antagonizing suppressive functions of MDSCs could enhance immune responses and virus control in chronic HCV infection.

## Introduction

Hepatitis C virus (HCV) infection is a major public health problem. Up to 85% of HCV infection become persistent, and may eventually lead to chronic liver diseases, including cirrhosis and hepatocellular carcinoma [1]. After infection is established, the interplay between the host and virus may lead to a unique disease progression pattern [2]. Many studies have demonstrated that the adaptive T cell immune response plays a major role both in controlling HCV infection and in contributing to hepatocellular damage [3]. The control of HCV infection requires a complex and coordinated interaction between innate and adaptive immune responses. However, the immune response to HCV is significantly impaired in patients with chronic infection. Although the recent development of direct acting antivirals (DAAs) improved HCV cure rates profoundly which targeted the HCV non-structural proteins [4], a prophylactic vaccine is not available. Therefore, a better understanding of the host immune response to HCV infection will be critical for HCV vaccine development.

It has been reported that HCV has evolved strategies to suppress the host immune response. HCV encodes several immunomodulatory proteins, such as HCV core protein (HCVc), envelop proteins and non-structural proteins to evade host immune defense. HCV-encoded proteins disable several innate immune signaling pathways by cleaving TRIF and MAVS [5]. Therefore it is important for improving therapy of HCV and for vaccine discovery to understand further the immune subversion mechanisms used by HCV.

The core protein is a major component of HCV nucleocapsids and shows various biological functions including immunomodulatory activity. It has been reported that HCVc can activate TLR2 in human monocytes, macrophages, Kupffer cells and regulatory T cells to induce an inflammatory cascade, including activation of IRAK-1 kinase, NF-kB, MAPK, and TNF-a production [6–10]. The HCVc protein also binds to and signals through the C1q complement receptor on macrophages and dendritic cells, leading to suppression of LPS-induced IL-12 expression [11]. In addition, HCVc blocks interferon signaling by inhibiting STAT-1 transcriptional activity [12], and activates STAT-3 transcriptional activity via an IL-6 autocrine pathway to impair inflammatory responses of monocytes/macrophage and dendritic cells [13].

Human monocytes circulate in the blood for 3–5 days, and are precursor cells of macrophages and dendritic cells. The function of monocytes is determined by immune modulatory interactions with HCV after viral infection [14]. Myeloid-Derived Suppressor Cells (MDSCs) are immature circulating myeloid cells with suppressive capability to host immune responses [15,16]. They were initially defined as CD14^+^ monocytes, which suppressed lymphocytes activation [17,18]. The MDSC-mediated suppression of T cell function is partly attributed to their increased indoleamine 2,3-dioxygenase (IDO) activity. Chronic lymphocytic leukemia (CLL) cells induces IDO^hi^ MDSCs from healthy donor monocytes, suggesting bidirectional crosstalks between tumor cells and monocytes [19]. The IDO enzyme catabolizes tryptophan to kynurenine. Recent studies have reported that the IDO1 expression in the liver of chronic HCV patients are up-regulated and their serum kynurenine/tryptophan ratio were increased [20,21]. IDO1 expression in the monocytes of chronic HCV patients is increased significantly but not in those from recovered patients [22]. However, signaling components involved in the up-regulation of IDO1 in monocytes of chronic HCV patients are so far largely unknown. More recent studies reveal that HCV induces the accumulation of MDSCs in human peripheral blood mononuclear cells (PBMCs), which may facilitate and maintain HCV persistent infection [23,24]. The mechanisms by which HCV induces MDSCs remains to be investigated.

More recently, we reported that HCVc induces monocytes to differentiate into monocytic myeloid-derived suppressor cells [25]. To further explore the mechanism by which HCV induces MDSCs, in the present study we performed an analysis of the transcriptome at first, and found that HCVc induces an MDSCs-like gene expression profile in monocytes. We thus hypothesized that HCV employs HCVc to induce MDSCs-like monocytes, which inhibit T cells proliferation by inducing CD4^+^Foxp3^+^ Tregs. Our results showed that both HCV virions and HCV core protein induced expression of IDO1, PD-L1 and IL-10, and significantly down-regulated HLA-DR expression in human monocytes. HCV-treated monocytes triggered CD4^+^Foxp3^+^ Treg cell expansion, and inhibited autologous CD4^+^ T cell activation via IDO1. We further found that HCV induced the MDSC-like suppressive monocytes through TLR2, PI3K, AKT and STAT3 signaling pathways. Finally, we validated that the levels of MDSCs and CD4^+^CD25^+^CD127^-^ Tregs in chronic HCV infected patients were higher than those in healthy individuals, and that a significant correlation existed between the frequency of MDSCs and CD4^+^CD25^+^CD127^-^ Tregs in patients with chronic HCV infection.

## Materials and Methods

### Ethics statement

The study was approved by the ethics committee of The First Hospital of Jilin University (No: 2015–215); written informed consent was obtained from all adult participants, and no children were involved in this study.

### Samples

Twenty-four treatment-naive chronic HCV-infected patients and sixteen healthy individuals were enrolled in this study (S1 Table). Venous blood was withdrawn for serum and peripheral blood collection. The date range in which human subjects’ data/samples were collected was February 2015 to February 2016.

### Reagents

Recombinant HCVc (aa 2–192 of the HCV polyprotein) and β-galactosidase were purchased from Meridian Life Science, Inc. (Saco, ME, USA) and the purifications were greater than 95%; Human TLR2 antibody, PD-L1 antibody and 1-methyl-DL-tryptophan (1-MT) were purchased from R&D; Human IL-10 antibody was purchased from eBioscience; LY-294002 hydrochloride and Stattic were obtained from Sigma-Aldrich.

### Detection of LPS contamination for HCVc

As described previously, endotoxin contamination of HCVc was detected using the QCL-1000^®^ chromogenic LAL endpoint assay (Cambrex, Cottonwood, AZ, USA) [10].

### Cell culture of HCV virion (HCVcc)

Cell cultures of HCV (HCVcc) production were obtained in JFH-Huh7.5 cells as described previously, and Huh7.5 cells (provided by Apath, LLC, Brooklyn, NY) were used as control [26]. In brief, JFH-Huh7.5 cells were cultured in 75T flasks in DMEM complete medium for about 5 days. Then the supernatants were harvested, cleared from cellular debris by filtration (0.45μm) and concentrated by ultracentrifugation. HCV RNA levels in HCVcc were determined using the COBAS AmpliPrep/COBAS TaqMan assay (Roche Molecular Diagnostics).

### Cell isolation

PBMCs were isolated based on the density gradient-based Ficoll-Paque. CD14^+^ monocytes and CD4^+^ T cells were purified using magnetic beads (Miltenyi Biotec). The purities of monocytes and CD4^+^ T cells were measured by flow cytometry and were always greater than 95%.

### Cell culture

Purified monocytes were cultured with increasing concentrations of HCVc (0.1,1.0,10 μg/ml) and HCVcc (10^4^, 10^5^, or 10^6^ copies/mL) in a 96 well plate containing 200 μl complete RPMI 1640 medium (1×10^6^ cells/well). Cells were collected for qRT-PCR and FACS analysis, and the supernatants were harvested for cytokine detection.

### RNA Isolation, cDNA library construction and illumina deep sequencing

Purified monocytes from three healthy donors were treated with 10 μg/ml HCVc for 10 hours in vitro. The following RNA extraction, cDNA library construction and deep sequencing on the Illumina sequencing platform (HiSeq^™^ 2500) were all completed in Shanghai OE Biotech Co., Ltd. (Shanghai, China).

### Flow cytometry

Peripheral blood cells staining was performed within 5 hours after blood collection. The antibodies used for fresh peripheral blood staining are shown as below: anti-HLA-DR-PE-CY7, anti-CD11b-APC, anti-CD33-V450, anti-CD14-FITC, anti-CD127-BV421, anti-CD3-FITC, anti-CD4-PERCP and anti-CD25-PE (all from BD Biosciences, USA). Matched isotype control antibodies were used as negative controls. Directly labeled antibodies were added on total blood and incubated for 30 min at 4°C in the dark, following by 10 minutes red blood cell lysis (BD Pharm Lyse^™^ lysing solution). Pure monocytes were stained for their surface phenotype (anti-PD-L1-BV421, anti-HLA-DR-PE-CY7, BD Biosciences) and intracellular IDO expression (anti-IDO-PE, ebioscience). BD LSRII Fortesa instrument was used to perform experiments and the data acquired were analyzed using FlowJo software.

### Measurement of tryptophan and kynuridine concentration

Kynuridine (kyn) release and tryptophan (trp) degradation were measured by liquid chromatography-mass spectrometry (LC-MS, Qtrap5500). Standard kyn and trp were purchased from Sigma-Aldrich.

### T cell proliferation assay

Pure monocytes were cultured with optimal concentrations of HCVc or HCVcc for two days. The HCVc- or HCVcc-treated monocytes were washed twice and co-cultured with autologous CD4^+^ T cells at a 1:1 ratio, which were stained with 5 mM CFSE (Molecular Probes). 10 μg/ml OKT3 (CD3 mAb, eBioscience) was precoated to plates overnight and anti-CD28 (5 μg/ml) antibody was then added to co-cultures as well. After co-cultured for 5 days, when clumps were visible, cells were collected and stained with anti-CD3-PE-CY7 and anti-CD4-Percp antibodies and intracellular anti-Foxp3-PE antibody. The proliferating CD4^+^ T cells and Foxp3 expression were characterized by flow cytometry. The supernatants were collected for IFN-γ ELISA assay. To investigate the involvement of selected molecules, blocking experiments were performed on day 0 of the co-culture by adding following inhibitor or antibodies: 1-MT (R&D, 0.5 mM), anti-IL-10 (eBioscience, 10 μg/ml), and anti-PD-L1 (R&D, 10 μg/ml). Control experiment were performed by using the isotype-matched mouse antibodies or vehicle media.

### Cytokine measurement

IFN-γ was quantified in supernatants using an ELISA kit (R&D Systems), the concentration of IL-10 in cell culture supernatant using an IL-10 ELISA kit (ebioscience) according to the manufacturer’s recommended protocol.

### RNA preparation and quantitative polymerase chain reaction

Total RNAs were extracted using RNeasymini kits (Qiagen), and cDNA were prepared using a cDNA Synthesis kit (Transgen). Messenger RNA quantifications were performed using real-time PCR (Roche SYBR Green). The specific primers used in our study are listed in S2 Table.

### Western blot analysis

Peripheral blood CD14^+^ monocytes were lysed in 1× RIPA buffer added with Protease/Phosphatase Inhibitor Cocktail (Cell signaling technology, Beverly, MA). Protein was quantified using the Bio-Rad protein assay (Bio-Rad, Hercules, CA, USA) at 595 nm, and separated by SDS-PAGE on a 10% gel. After transferred to a Nitrocellulose membrane, the membrane was blocked in PBS supplemented with 5% Bovine Serum Albumin (BSA, Amresco) and then incubated with primary antibodies (1:1000) and the following secondary antibodies (1:2000), which were purchased from CST. The target proteins were assessed using ECL Plus reagent (GE Healthcare).

### Statistical analysis

The data we analyzed were compared using unpaired t tests to determine the statistical significance. All statistical analysis were performed using GraphPad Prism 5 software.

## Results

### HCVc induces a distinctive gene expression profile in CD14^+^ monocytes

**Fig 1:**
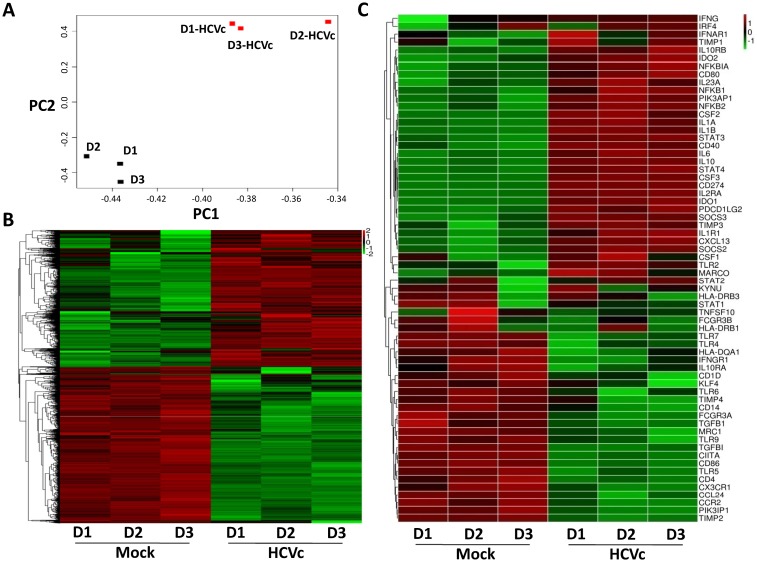
HCVc induces a distinctive gene expression profile in CD14^+^ monocytes. Monocytes were purified from three healthy donors by CD14^+^ microbeads, and cultured with HCVc for 12 hours. RNAseq was performed to analyze the change in transcriptomic profiles. (A) PCA plot representing differential clustering of monocytes treated with HCVc and controls based on mRNA expression profiles. (B) Self-organizing heat-map of the different expression genes with p-value<0.05 across all samples, showing different expression profiles in monocytes from HCVc-treated compared to controls. (C) The heat map shows the relative expression levels of 64 genes (row) of immune responses from monocytes and macrophages. The color scale indicates the degree of relative expression of immune genes (deep red, high expression; deep green, low expression). Abbreviation: RNAseq, RNA sequencing; PCA, Principal Component Analysis.

Our previous work reported that HCVc induced CD14^+^ monocytes to differentiate into monocytic (Mo)-MDSCs [25]. We performed RNAseq to investigate if HCVc induced a distinctive gene expression profile in CD14^+^ monocytes. Principal Component Analysis (PCA) showed that the gene expression profiles of HCVc-treated and mock monocytes clustered distinctively (Fig 1A). Self-organizing heat-maps confirmed that HCVc-treated and mock monocytes displayed different gene expression profiles (Fig 1B). Among the most differentially expressed genes, we observed that HCVc induced monocytes a suppressive gene expression profile (i.e. up-regulation of IDO1, IDO2, CD274, IL-10, IL-10RB, SOCS2, SOCS3, and down-regulation of HLA-DR, IL-10RA, CD86), and a distinctive signaling pathway activation (i.e. up-regulation of STAT3, PIK3AP1 and down-regulation of STAT1, PIK3IP1), compared with controls (Fig 1C).

### HCVc and HCVcc induce monocytes with suppressive characteristics in vitro

**Fig 2:**
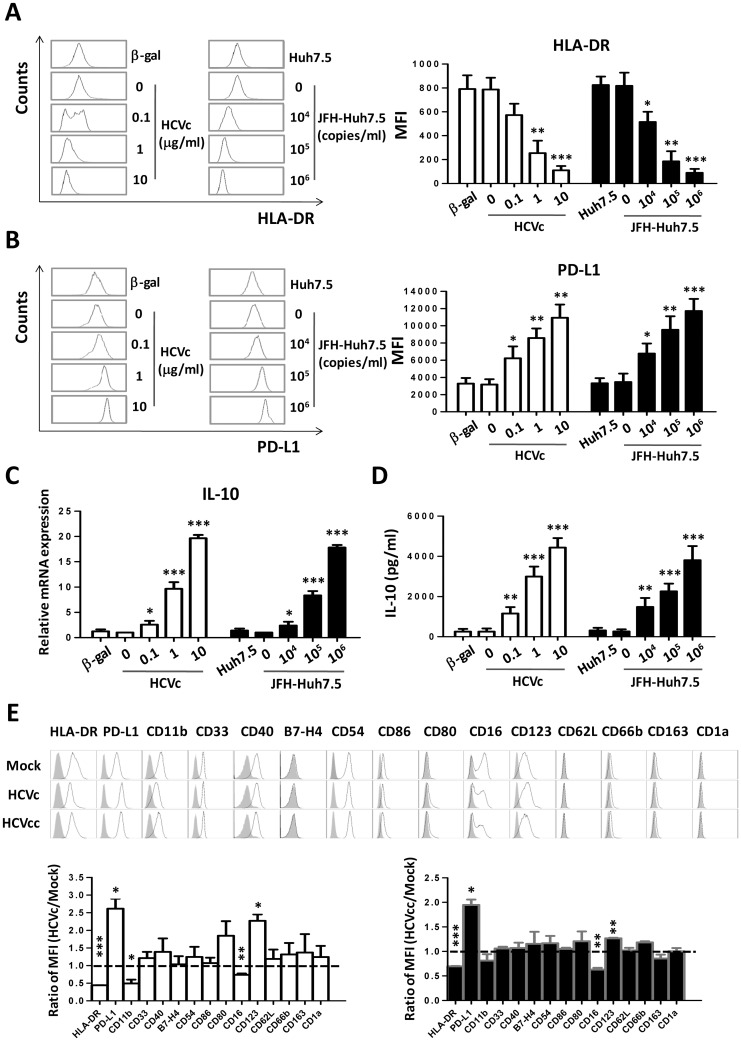
Both HCVc and HCVcc induce suppressive monocytes in vitro. Purified monocytes from healthy HCV-negative blood donors were cultured for two days in the presence of increasing concentrations of HCVc (0.1, 1.0, 10.0 μg/ml) or HCVcc (10^4^, 10^5^,10^6^ HCV genome copies/ml). HLA-DR (A) and PD-L1 (B) expression was analyzed by flow cytometry. (C) The mRNA expression of IL-10 was determined by qRT-PCR. (D) IL-10 production was assayed with ELISA. (E) The surface marker expression profile of monocytes was analyzed by flow cytometry. One of five representative experiments is shown. MFI is normalized as the ratio of HCVc/mock and HCVcc/mock for surface marker expression profile. Abbreviation: MFI, Mean Fluorescence intensity.

In order to strengthen the conclusion that HCVc induced in monocytes a suppressive gene expression profile, freshly purified monocytes from healthy HCV-negative blood donors were cultured for two days in the presence of increasing concentrations of HCV core protein (HCVc) or cell culture-derived HCV (HCVcc). Our results showed that the down-regulation of HLA-DR expression was induced by HCVc and HCVcc, with significant decrease at 1μg/ml HCVc (MFI 255±59, p = 0.0029) and 10^4^ copies/ml HCVcc (MFI 515±49, p = 0.0194) as compared with medium (MFI 787±57 or 818±63) (Fig 2A) as previously reported [23]. We also observed that increased PD-L1 expression, which is a key functional molecule in suppressing T cell proliferation and activation [27–30], was significantly induced by both of HCVc and HCVcc in a dose dependent manner (Fig 2B). Cells were collected at 10 hours for assay of IL-10 mRNA levels by qRT-PCR, the results showed that both HCVc and HCVcc caused a dose-dependent increase in IL-10 mRNA expression (Fig 2C) in monocytes, which was consistent with the transcriptomic profiles by RNAseq. The cell supernatants were collected at 48 hours, and IL-10 production was assayed by ELISA. Both HCVc and HCVcc induced a dose-dependent and significant increase of IL-10 production (Fig 2D). These data suggest that HCV may employ HCVc to induce IL-10 expression and production, as well as to up-regulate PD-L1, and down-regulate HLA-DR in monocytes.

To further characterize HCV-induced suppressive monocytes, we analyzed their surface marker expression profile. A representative result was shown in Fig 2E. Using monocytes cultured with tissue culture medium alone (mock) as control, HCVc and HCVcc significantly induced HLA-DR down-regulation and PD-L1 up-regulation in monocytes as above. In addition, a decreased expression level of CD11b and CD16, and an increased CD123 expression, were observed on monocytes cultured with either HCVc or HCVcc. There was no difference with respect to the expression level of CD33, CD54, CD40, CD80, CD86, B7H4, CD66b, CD62L, CD1a and CD163 on HCVc- and HCVcc-induced or control monocytes.

### HCVc and HCVcc induce IDO1 expression in monocytes

**Fig 3:**
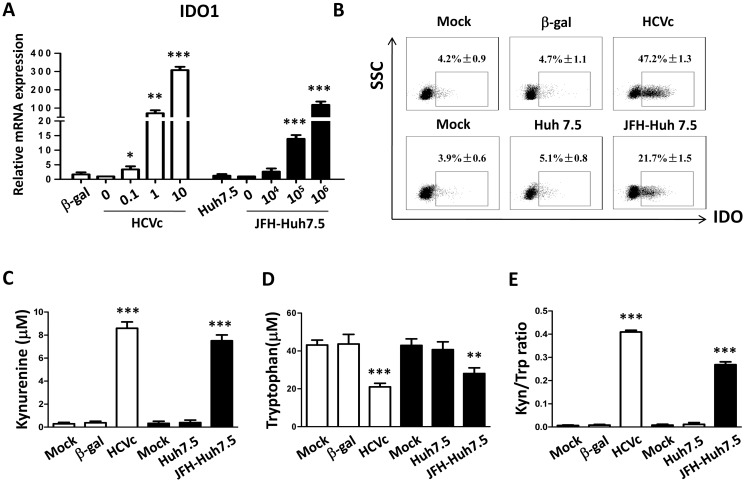
HCVc and HCVcc induce IDO1 expression, and produce kynurenine (Kyn) and degradate tryptophan (Trp) in monocytes. Purified monocytes from healthy HCV-negative blood donors were cultured with HCVc or HCVcc. Cells were collected at 10 hours for measuring IDO1 mRNA levels by qRT-PCR (A). Intracellular staining was performed for IDO1 expression (B). The cell culture supernatants were collected, and the concentrations of Kyn and Trp were examined by liquid chromatography-mass spectrometry. The concentrations of Kyn (C) and Trp (D) are shown, and E shows the ratio of Kyn/Trp. The bars represent the standard error of the mean. Abbreviation: Kyn, kynurenine; Trp, tryptophan; IDO1, indoleamine 2,3-dioxygenase 1.

IDO1 is the rate-limiting enzyme that catalyzes tryptophan (Trp) degradation through the kynurenine (Kyn) pathway [31]. Elevated tryptophan catabolism leads to Trp starvation of T cells, and subsequently to limit T cell proliferation and activation [32]. Moreover, IDO1 can also enhance immunosuppression mediated by regulatory T cells (Tregs) [33]. We further tested whether HCVc and HCVcc induce IDO1 expression to produce Kyn and degrade Trp in monocytes. Purified monocytes from healthy HCV-negative blood donors were cultured with HCVc or HCVcc. Cells were collected at 10 hours for measuring IDO1 mRNA levels by qRT-PCR. The result showed that both HCVc and HCVcc caused a dose-dependent increase in IDO1 mRNA expression in monocytes (Fig 3A). Intracellular staining of IDO1 proteins in monocytes showed that HCVc or HCVcc induced 5–10 fold up-regulation above control (Fig 3B, HCVc: 47.2%±1.3, HCVcc: 21.7%±1.5 vs control: 4.2%±0.9 or 3.9%±0.6). The cell culture supernatants were obtained and Kyn and Trp levels were examined by LC-MS. Both HCVc and HCVcc induced monocytes to release 10 fold higher Kyn and 2 fold lower Trp than controls (Fig 3C, 3D and 3E). These results indicate that HCV induced IDO1 expression in monocytes to catalyze Trp degradation and Kyn accumulation.

### HCV-induced suppressive monocytes inhibit autologous CD4^+^ T cell activation, associated with CD4^+^Foxp3^+^ Treg expansion in an IDO1-dependent manner

**Fig 4:**
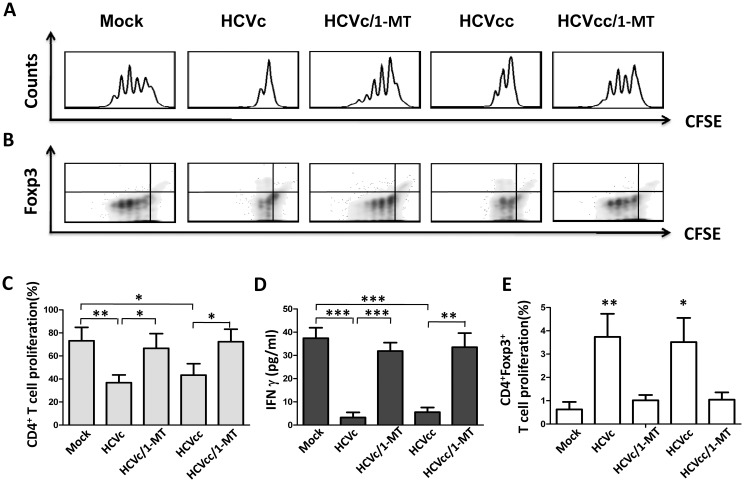
HCVc and HCVcc induce suppressive monocytes to inhibit autologous CD4^+^ T cell activation and expand CD4^+^Foxp3^+^ Tregs in an IDO1-dependent manner. CFSE-labeled CD4^+^ T cells were stimulated with pre-coated OKT3 (CD3 mAb) and CD28 antibody, and co-cultured with HCVc- and HCVcc-treated autologous monocytes in the absence or presence of 1-MT. Foxp3 expression and proliferation of the autologous CD4^+^ T cell were evaluated by intracellular staining and flow cytometry. One of five representative experiments is shown as histogram (A) and contour (B). The proliferation of CD4^+^ T cells (C) and CD4^+^Foxp3^+^ T cells (E) are statistically analyzed from five experiments. The supernatants were collected and assayed by ELISA for IFN γ production (D). The bars represent the standard error of the mean. Abbreviation: 1-MT, 1-methyl-DL-tryptophan.

We next investigated whether HCVc-induced suppressive monocytes inhibit T cell activation. CFSE-labeled autologous CD4^+^ T cells were stimulated with pre-coated OKT3 (CD3 mAb) and CD28 antibody, and co-cultured with control or HCVc- and HCVcc-treated monocytes. Both HCVc and HCVcc-treated monocytes significantly suppressed autologous CD4^+^ T cell proliferation (Fig 4A, 4B and 4C) and IFN-γ secretion (Fig 4D). In addition, HCVc- and HCVcc-treated monocytes significantly induced Foxp3 expression on CD4^+^ T cells and Foxp3^+^CD4^+^ T cell proliferation (Fig 4B and 4E), compared with untreated monocytes when cultured with autologous CD4^+^ T cells. Next, the suppressive effect of the candidate molecules was tested in the co-cultures of monocytes and CD4^+^ T cells, by using specific inhibitors and antibodies against IDO1, PD-L1, and IL-10. IDO inhibitor (1-methyl-DL-tryptophan, 1-MT), anti-PD-L1 or anti-IL-10 antibody were added to HCVc and HCVcc-treated monocytes co-cultured with autologous CD4^+^ T cells. We observed that 1-MT abolished the suppressive ability of HCVc- and HCVcc-treated monocytes on CD4^+^ T cell proliferation (Fig 4A, 4B and 4C) and IFN-γ secretion (Fig 4D), and dampened Foxp3^+^CD4^+^ T cell expansion induced by HCVc and HCVcc-treated monocytes (Fig 4B and 4E). However, anti-PD-L1 and anti-IL-10 antibodies showed no significant effect (S1 Fig). In addition, HCVc- and HCVcc-treated monocytes were co-cultured with autologous T cells, we found that HCVc- and HCVcc-treated monocytes also exhibited their suppressive ability on CD8^+^ T cell proliferation (S2 Fig).

### HCVc and HCVcc induce suppressive monocytes via TLR2/PI3K/AKT/STAT3 signaling pathways

**Fig 5:**
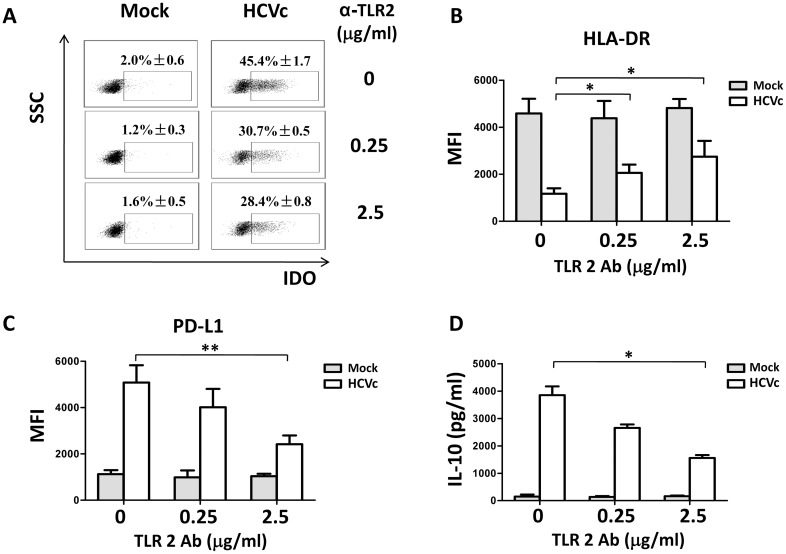
HCVc induces suppressive monocytes through engagement with TLR2. Purified monocytes were pretreated with anti-TLR2 antibody (0.25 and 2.5 μg/ml), and then stimulated with HCVc for two days. Cells were collected for the analysis of IDO1 (A), HLA-DR (B), and PD-L1 (C) expression by flow cytometry. The supernatants were collected for detecting IL-10 by ELISA (D).

We have reported that HCVc induces TLR2-mediated activation of monocytes [10]. We thus tested if HCVc-induced suppressive monocytes were similarly mediated through engagement with TLR2. Purified monocytes were pretreated with different concentrations of anti-TLR2 antibody, and then treated with HCVc for two days. As expected, blockade of HCVc/TLR2 engagement with the anti-TLR2 antibody inhibited HCVc-induced expression of IDO1 (Fig 5A), PD-L1 (Fig 5C), and IL-10 (Fig 5D), and restored partially HLA-DR expression (Fig 5B).

**Fig 6:**
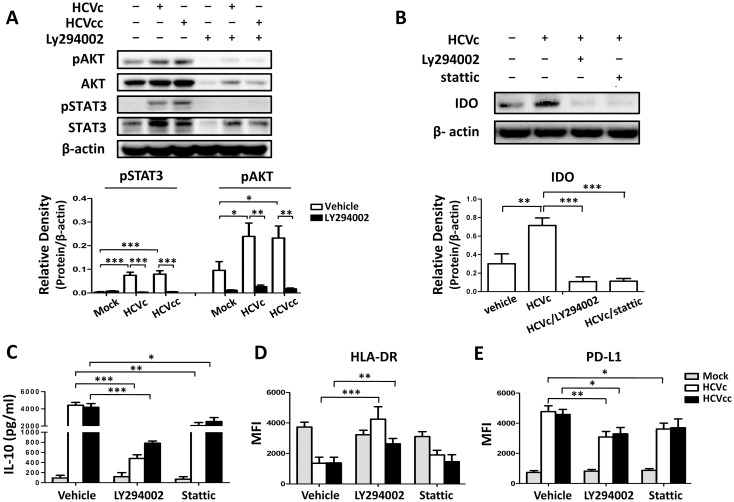
PI3K and STAT3 are involved in HCVc- and HCVcc-induced suppressive monocytes. Purified monocytes from healthy HCV-negative blood donors were cultured with (10.0 μg/ml) or HCVcc (10^6^ copies/ml) in the presence or absence of PI3K inhibitor (LY294002) or STAT3 inhibitor (stattic). Western blots were performed for AKT/AKT phosphorylation and STAT3/ STAT3 phosphorylation (A), and IDO1 expression (B). β actin was used as loading control. The supernatants were collected for IL-10 detection by ELISA (C). Cells were collected for the analysis of HLA-DR (D) and PD-L1 (E) expression by flow cytometry.

To further identify the mechanism(s) responsible for HCVc- and HCVcc-induced suppressive monocytes, we examined the activation status of important signaling and transcription factors which influence monocyte activation and function following exposure to HCVc and HCVcc. STAT3 and AKT phosphorylation levels were increased following HCVc and HCVcc treatment, and LY2904002 (an inhibitor of PI3K) inhibited the HCVc- and HCVcc- induced STAT3 and AKT phosphorylation (Fig 6A). These results clearly indicate that HCVc- and HCVcc-mediated signaling requires the activation of PI3K/AKT for STAT3 phosphorylation. We further validated that the CD14^+^ monocytes from HCV patients had higher levels of AKT and STAT3 phosphorylation than healthy controls (S3 Fig).

We next sought to determine whether HCVc- and HCVcc-mediated signaling pathway through PI3K/AKT/STAT3 resulted in monocytes with suppressive characteristics. Inhibitors of PI3K and STAT3 abolished HCVc-induced expression of IDO1 (Fig 6B), of IL-10 production (Fig 6C), and of PD-L1 up-regulation (Fig 6E), and restored expression of HLA-DR (Fig 6D). These results suggest that HCVc and HCVcc induce MDSC-like monocytes through the PI3K/AKT/STAT3 signaling pathway.

### Peripheral monocytes from patients with chronic HCV infection display MDSC-like suppressive characteristics, correlated with a significant increase in CD14^+^ MDSCs and CD4^+^ Tregs in chronic HCV infected patients compared to healthy individuals

**Fig 7:**
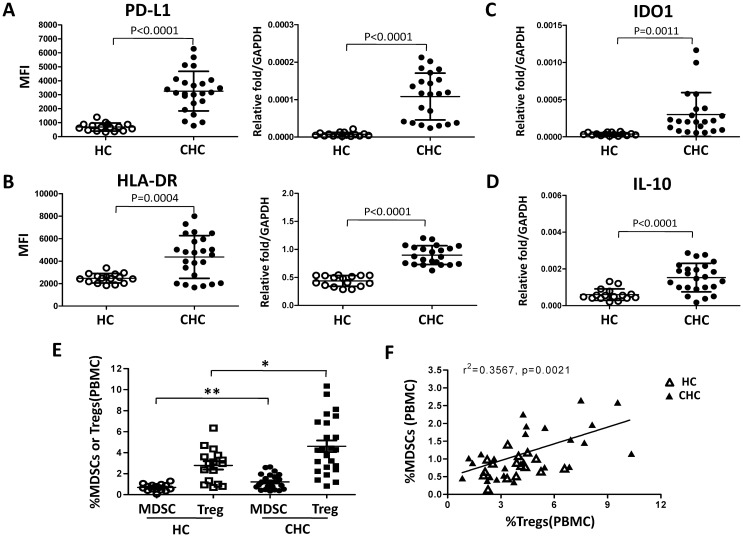
Patients with chronic HCV infection display higher frequency of monocytic MDSCs and CD4^+^CD25^+^CD127^-^ Tregs. PBMCs were isolated from healthy individuals (n = 16) and chronic hepatitis C patients (n = 24). PD-L1 (A) and HLA-DR (B) expression of monocytes was analyzed by flow cytometry. Monocytes from healthy individuals and patients with HCV infection were purified, and expression of IDO1 (C), PD-L1 (A), HLA-DR (B), and IL-10 (D) mRNA in monocytes was determined by qRT-PCR. Monocytic MDSCs and CD4^+^CD25^+^CD127^-^ Tregs were characterized in peripheral blood of patients with chronic HCV infection and healthy individuals by flow cytometry. E shows statistical analysis, and F shows the correlation between the frequency of MDSCs and CD4^+^CD25^+^CD127^-^ Tregs in chronic hepatitis C patients. Abbreviation: PBMCs, peripheral blood mononuclear cells; HC, healthy control; CHC, chronic hepatitis C patients.

Peripheral blood mononuclear cells (PBMCs) were isolated from healthy individuals (n = 16) and chronic HCV infected patients (n = 24). We first analyzed PD-L1 and HLA-DR expression on peripheral blood monocytes of the two group individuals. Peripheral blood monocytes were identified by their size and granularity in forward and side scatter plots and CD14 expression. The results were shown in Fig 7A and 7B. The expression levels of PD-L1 and HLA-DR on monocytes from patients with HCV infection were significantly higher than those from healthy individuals (PD-L1 MFI: 3258±289 vs 690±71.3, p<0.0001; HLA-DR MFI: 4363±387.5 vs 2471±106.1, p = 0.0004).

We then purified peripheral blood monocytes from healthy controls and chronic HCV infected patients by using CD14 microbeads. The expression levels of HLA-DR, PD-L1, IL-10, and IDO1 were determined by qRT-PCR. We found that monocytes from chronic HCV infected patients expressed higher levels of IDO1 (p = 0.0011), PD-L1 (p<0.0001), HLA-DR (p<0.0001), and IL-10 (p<0.0001) than healthy controls (Fig 7A, 7B, 7C and 7D). Finally, we investigated the characteristics of monocytic MDSCs and CD4^+^CD25^+^CD127^-^ Tregs in peripheral blood of patients with chronic HCV infection and healthy individuals. Peripheral blood from twenty-four treatment-naive chronic HCV-infected patients and sixteen healthy individuals were collected, and monocytic MDSCs and CD4^+^CD25^+^CD127^-^ Tregs were identified according to the gating strategy shown in S4 Fig. Patients with chronic HCV infection showed significant increases in the frequencies of MDSCs and CD4^+^CD25^+^CD127^-^ Tregs compared to healthy individuals (Fig 7E), and a positive correlation existed between the frequency of MDSCs and that of CD4^+^CD25^+^CD127^-^ Tregs (Fig 7F), as well as positive correlation of HCV RNA level or HCVc protein level with the percentages of CD14^+^ MDSCs and CD4^+^CD25^+^CD127^-^ Tregs in peripheral blood of chronic HCV patients was shown in S5 Fig. These results support the hypothesis that HCV induces MDSCs-like monocytes that inhibit T cell proliferation by inducing or expanding CD4^+^Foxp3^+^ Tregs.

## Discussion

HCV viruses and core protein (HCVc) are detected in the blood of HCV infected patients [34,35]. Our previous study has shown that the serum level of HCVc is positively correlated with HCV RNA levels, indicating that HCVc concentration represents a stable and reliable marker of HCV viral replication [9]. It has been reported that HCVc is not only an HCV encoded RNA-binding viral capsid protein, but also an important protein involving in immunomodulation [36,37]. HCVc binds and signals through TLR2, leading to cytokine production and immune dysregulation of monocytes [7]. Our recent studies have reported that HCVc induces monocytes to differentiate into monocytic (Mo)-MDSCs [25]. In this study, we found that HCVc induced monocytes with a suppressive gene expression profile, including up-regulation of IDO1, CD274, IL-10, STAT3, PIK3AP1 and down-regulation of HLA-DR, CD86, STAT1, PIK3IP1 compared with controls. Therefore, we conclude that HCV exploited HCVc to induce suppressive monocytes. Our results showed here that both HCVc and HCVcc significantly up-regulated PD-L1, IL-10 expression and production, and down-regulated HLA-DR expression on monocytes. In addition, decreased CD11b and CD16 expression levels were observed on monocytes treated with HCVc or HCVcc (Fig 2). Moreover, both HCVc and HCVcc induced IDO1 expression in monocytes to catalyze the Trp degradation and Kyn accumulation (Fig 3). Furthermore, we found that blockade of HCVc interaction with TLR2 inhibited HCVc-induced PD-L1 and IDO1 expression, and IL-10 production (Fig 5). These results indicate that HCV induces suppressive monocytes by HCVc engagement with TLR2 [10].

It has been reported that T cell immune function in chronic HCV infection is significantly impaired [38]. HCV exploits active immunosuppressive strategies to interfere with antigen-presenting cells, such as DC and macrophages to impair the function of adaptive immune effector cells [13]. Regulatory T cells (Tregs) are increased in chronic HCV infected patients, which may contribute to the sustained suppression of HCV-specific T-cell responses and persistant HCV infection. In 87 chronic HCV-infected patients, our recent study indicated that the level of HCVc showed a significant correlation with that of CD4^+^CD25^+^Foxp3^+^ Tregs [9]. In this study, our results showed that both HCVcc- and HCVc-treated monocytes expanded CD4^+^Foxp3^+^ Tregs, and inhibited autologous CD4^+^ and CD8^+^ T cell activation. Moreover, 1-methyl-DL-tryptophan (1-MT, a specific inhibitor of IDO1) suppressed CD4^+^Foxp3^+^ T cell expansion triggered by HCVc- and HCVcc-treated monocytes, and abolished the ability of HCVc- and HCVcc-treated monocytes to suppress CD4^+^ T cell activation (Fig 4). Therefore, these results further demonstrate that HCV employed HCVc to increase CD4^+^CD25^+^Foxp3^+^ regulatory T cells and inhibit T cell immune response by inducing suppressive monocytes. Recent studies have demonstrated a crucial role of Indoleamine 2,3-dioxygenase (IDO) in the induction of immune tolerance during infection, pregnancy, transplantation, autoimmunity, and cancers [39,40]. IDO induces Tregs and inhibits T-cell activation through Trp starvation and/or the accumulation of Kyn [41,42]. Our results showed that both HCVc and HCVcc promoted monocyte-mediated Trp degradation and induced the accumulation of Kyn (Fig 3). An increased Kyn/Trp ratio in the cell culture supernatant of monocytes treated with HCVc or HCVcc was in accordance with the elevated IDO1 expression. The induction of IDO1 expression is likely a strategy of HCV to suppress the host immunity and promote HCV persistence. Our study indicate that the IDO1 inhibitor 1-MT effectively restores CD4^+^ T cell proliferation co-cultured with HCVc- or HCVcc-treated monocytes. These results are consistent with previous studies, which report that HCV induces the accumulation of MDSCs leading to suppressing autologous T cells in an ROS-dependent manner [23]. MDSCs clearly work in more than one way, but our results imply that the use of a selective IDO1 inhibitor might be promising as a treatment of treating HCV infection.

Signal transducer and activator of transcription (STAT) protein 3 is important in the regulation of inflammatory responses by APCs. In monocytes, STAT3 exerts a critical function in limiting excessive inflammatory responses [43]. Our previous report demonstrated that the expression of PD-L1 (induced by TLR2 and TLR4) and TRAIL (induced by TLR3) on human Kupffer cells depended on phosphatidylinositol-3-kinase (PI3K) activity, revealing the cross talk between the MyD88 and toll-interleukin 1 receptor-domain-containing adapter-inducing IFN (TRIF) [7]. It has been reported that an important role for extracellular HCVc in the activation of STAT3 in human monocytes depends upon the PI3K/AKT pathway [13]. In agreement with it, our results showed that both HCVc and HCVcc induced STAT3 and AKT phosphorylations in monocytes. We also observed that the peripheral monocytes from HCV patients had higher levels of AKT and STAT3 phosphorylation than healthy controls. In addition, LY294002 (PI3K inhibitor) inhibited not only HCVc- and HCVcc-induced AKT phosphorylations, but also STAT3 phosphorylations. We further found that HCVc-induced IDO1 expression and IL-10 production were inhibited by either PI3K or STAT3 inhibitors, and the inhibitors of PI3K and STAT3 also reversed the inhibition of HLA-DR expression by HCVc and HCVcc (Fig 6). A previous study has reported that PI3K-specific inhibition or shRNA knockdown diminishes IFNγ-induced IDO production [44]. These results suggest that HCV employs HCVc to induce MDSC-like suppressive monocytes through the TLR2/PI3K/AKT/STAT3 signaling pathway.

Monocytes provide the first line of defense against pathogens, and play an important role in immune surveillance and immunoregulation during HCV infection. Recent reports show that monocytes are involved in HCV persistent infection through expressing suppressive molecules, such as PD-L1 and IDO1, and delivering negative signals to T cells [22,45]. The upregulation of PD-L1 in monocytes is associated with impaired HCV-specific T cell responses, and the frequency IFN-γ-producing HCV-specific T cells are significantly enhanced when PD-L1 is inhibited [46]. Over-expression of IDO1 inhibits T cell activation by catabolizing the initial rate-limiting reaction of Trp [47]. In the present study, we found that the expression levels of PD-L1, IL-10 and IDO1 on monocytes from patients with HCV infection were significantly higher than those from healthy individuals, which was consistent with HCVc- and HCVcc-treated monocytes in vitro. However, the enhanced expression of HLA-DR in monocytes was also observed in patients with HCV infection, indicating persistent activation of monocytes in HCV-infected patients.

Myeloid-derived suppressor cells (MDSCs) are characterized by an aberrant myeloid phenotype and the ability to suppress immue responses. Human MDSCs are dichotomized into granulocytic and monocytic subsets. They exert their suppressive function through a plethora of mechanisms, including production of reactive oxygen species (ROS), immune-modulating cytokines (eg, IL-10), cyclooxygenase-2 (COX2), arginase-1 (ARG1), and indoleamine-2,3-dioxygenase (IDO) [48]. Based on our data and literatures, therefore, we suppose that HCVc and HCVcc induce monocytes differentiation to monocytic MDSCs, which express inhibitory molecules PD-L1, IDO1, IL-10, and suppress autologous CD4^+^ T cell activation. Consistently, we observed in this study that the expression levels of PD-L1, IL-10 and IDO1 on monocytes from patients with HCV infection were significantly higher than those from healthy individuals. Finally, we characterized monocytic MDSCs and CD4^+^CD25^+^CD127^-^ Tregs in peripheral blood of patients with chronic HCV infection and healthy individuals according to their specific markers, and found that both of the frequencies of MDSCs and CD4^+^CD25^+^CD127^-^ Tregs in chronic HCV infection were significantly higher than healthy individuals, which was consistent with some recent studies [24,49,50]. We also found the correlation between the frequency of MDSCs and CD4^+^CD25^+^CD127^-^ Tregs (Fig 7), and the correlation of HCV RNA level or HCVc protein level with the percentages of CD14^+^ MDSCs and CD4^+^CD25^+^CD127^-^ Tregs in peripheral blood of chronic HCV patients, respectively.

In sum, our results indicate that HCV employed HCVc to induce MDSC-like monocytes via TLR2/PI3K/AKT/STAT3 signaling pathway. These MDSC-like monocytes inhibited CD4^+^ T cell activation and expanded CD4^+^CD25^+^Foxp3^+^ Tregs by IDO1-induced Trp degradation and the accumulation of Kyn. Moreover, we validated that peripheral monocytes from patients with chronic HCV infection displayed MDSCs characteristic in comparison to monocytes from healthy individuals, and the correlation between the frequency of MDSCs and CD4^+^CD25^+^CD127^-^ Tregs in patients with chronic HCV infection. Taken together, our study identified the critical upstream signals for the induction of IDO1 in monocytes stimulated by hepatitis C virus that could be manipulated therapeutically to enhance or decrease their immunosuppressive function for HCV treatment. It will be of interest to test whether antagonizing suppressive functions of MDSCs could represent a mean for enhancing immune responses in chronic HCV infection.

## Supporting Information

S1 Dataset Mean Fluorescence intensity of PD-L1 on monocytes from healthy individuals and chronic hepatitis C patients analyzed by flow cytometry. (XLSX)

S1 Fig HCVc induce suppressive monocytes to inhibit autologous CD4^+^ T cell proliferation and expand CD4^+^Foxp3^+^ Tregs in an IDO1-dependent manner, while PD-L1 and IL-10 show no significant effect. (TIF)

S2 Fig HCVc and HCVcc induce suppressive monocytes to inhibit autologous CD8^+^ T cell proliferation. Purified monocytes from healthy HCV-negative blood donors were cultured with HCVc or HCVcc for 2 days. The HCVc- or HCVcc-treated monocytes were washed twice and co-cultured with the purified autologous CD3^+^ T cells (ratio = 1:2 and 1:1), which were stained with 5 mM CFSE and stimulated with pre-coated OKT3 (CD3 mAb) and CD28 antibody. After 5 days co-culture, the proliferating of the autologous CD3^+^CD8^+^ T cells were characterized by flow cytometry (A) and statistically analyzed from three experiments (B).
(TIF)

S3 Fig Flow cytometric analysis of Akt (pS473) and Stat3 (pY705) levels in the peripheral CD14^+^ monocytes of HCV patients. PBMCs were isolated from healthy individuals (unshaded, n = 3) and treatment-naive chronic hepatitis C patients (shaded, n = 5). Cells were stained with anti-CD14-APC-Cy7 antibody, and then fixed for 10 min at 37°C using BD Phosflow^™^ Fix Buffer I (Cat. No. 557870), permeabilized in BD Phosflow^™^ Perm Buffer III (Cat. No. 558050) on ice for 30 min. Cells were washed twice and stained with PE-CF594 Mouse Anti-Akt (pS473) antibody and Alexa Fluor 647 Mouse Anti-Stat3 (pY705) antibody for 30 min at room temperature. CD14^+^ monocytes were analyzed for AKT phosphorylation (A) and STAT3 phosphorylation status (B). Abbreviation: PBMCs, peripheral blood mononuclear cells; HC, healthy control; CHC, chronic hepatitis C patients; MFI, Mean Fluorescence intensity.
(TIF)

S4 Fig Gating strategy for monocytic MDSCs and CD4^+^CD25^+^CD127^-^ Tregs. (TIF)

S5 Fig Correlation of HCV RNA level or HCVc protein level with the percentages of CD14^+^ MDSCs (A) and CD4^+^CD25^+^CD127^-^ Tregs (B) in peripheral blood of 24 chronic HCV patients. (TIF)

S6 Fig Rough uncut image for Fig 6. (TIF)

S1 Table Characteristics of hepatitis C patients and healthy controls. (DOCX)

S2 Table Primer sequences for qRT-PCR. (DOCX)
