# Supplementary figures and images for: Hepatitis C Virus Induces MDSCs-Like Monocytes through TLR2/PI3K/AKT/STAT3 Signaling
Source: PLoS One. 2017 Jan 23;12(1):e0170516. doi: 10.1371/journal.pone.0170516 (PMC5256909; doi:10.1371/journal.pone.0170516)

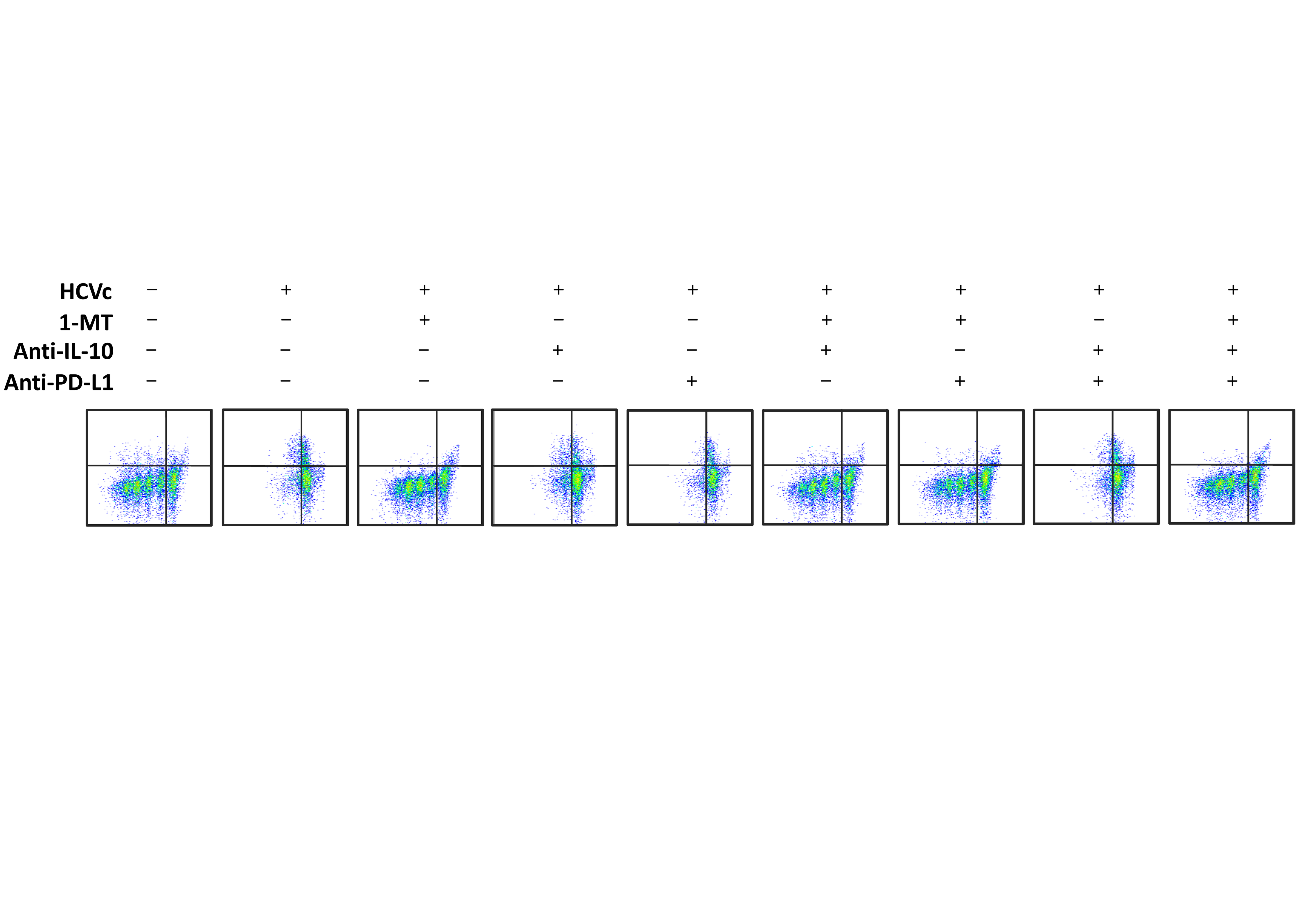

Supplement: S1 Fig — (TIF) [file pone.0170516.s002.tif]

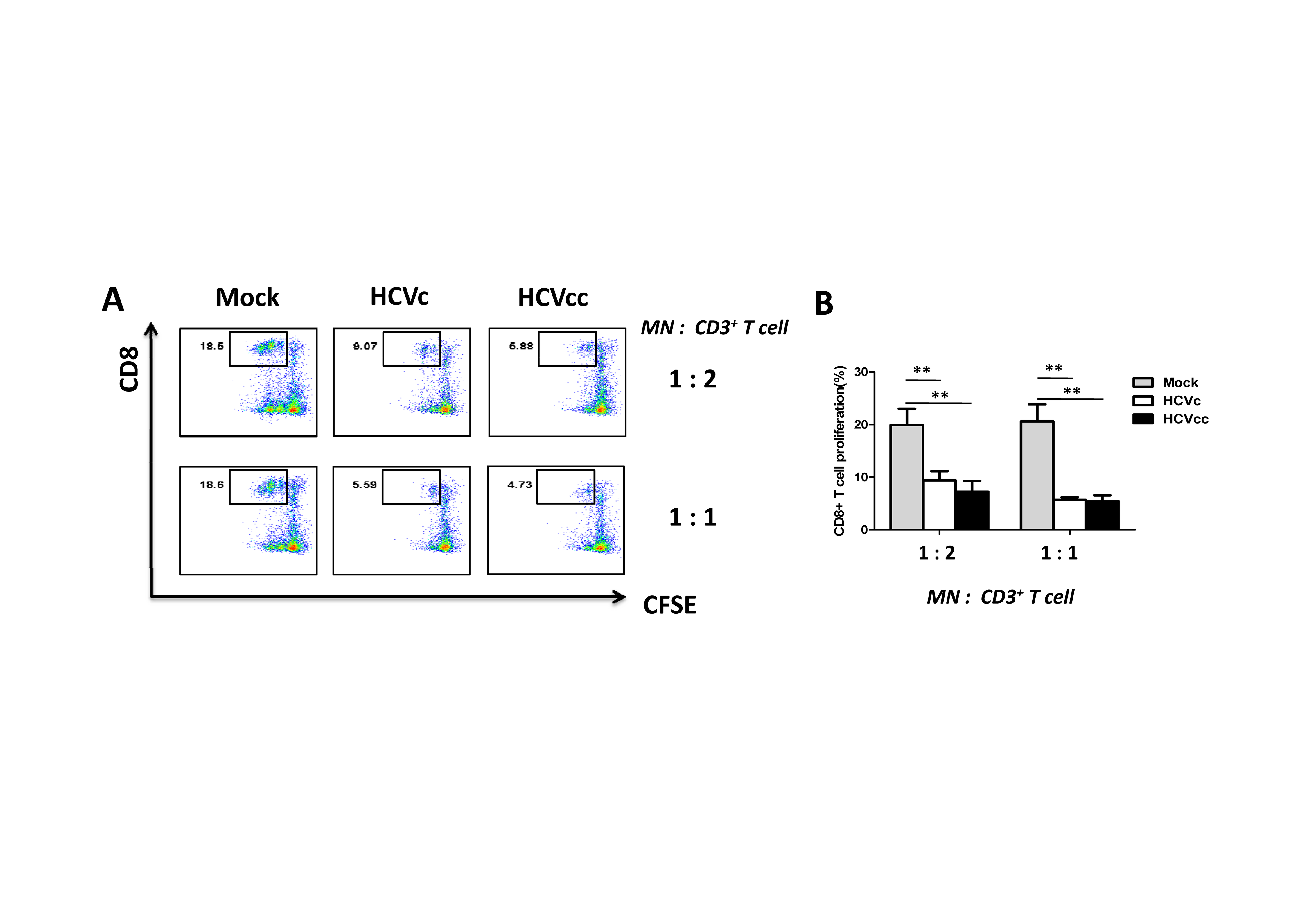

Supplement: S2 Fig — Purified monocytes from healthy HCV-negative blood donors were cultured with HCVc or HCVcc for 2 days. The HCVc- or HCVcc-treated monocytes were washed twice and co-cultured with the purified autologous CD3+ T cells (ratio = 1:2 and 1:1), which were stained with 5 mM CFSE and stimulated with pre-coated OKT3 (CD3 mAb) and CD28 antibody. After 5 days co-culture, the proliferating of the autologous CD3+CD8+ T cells were characterized by flow cytometry (A) and statistically analyzed from three experiments (B). (TIF) [file pone.0170516.s003.tif]

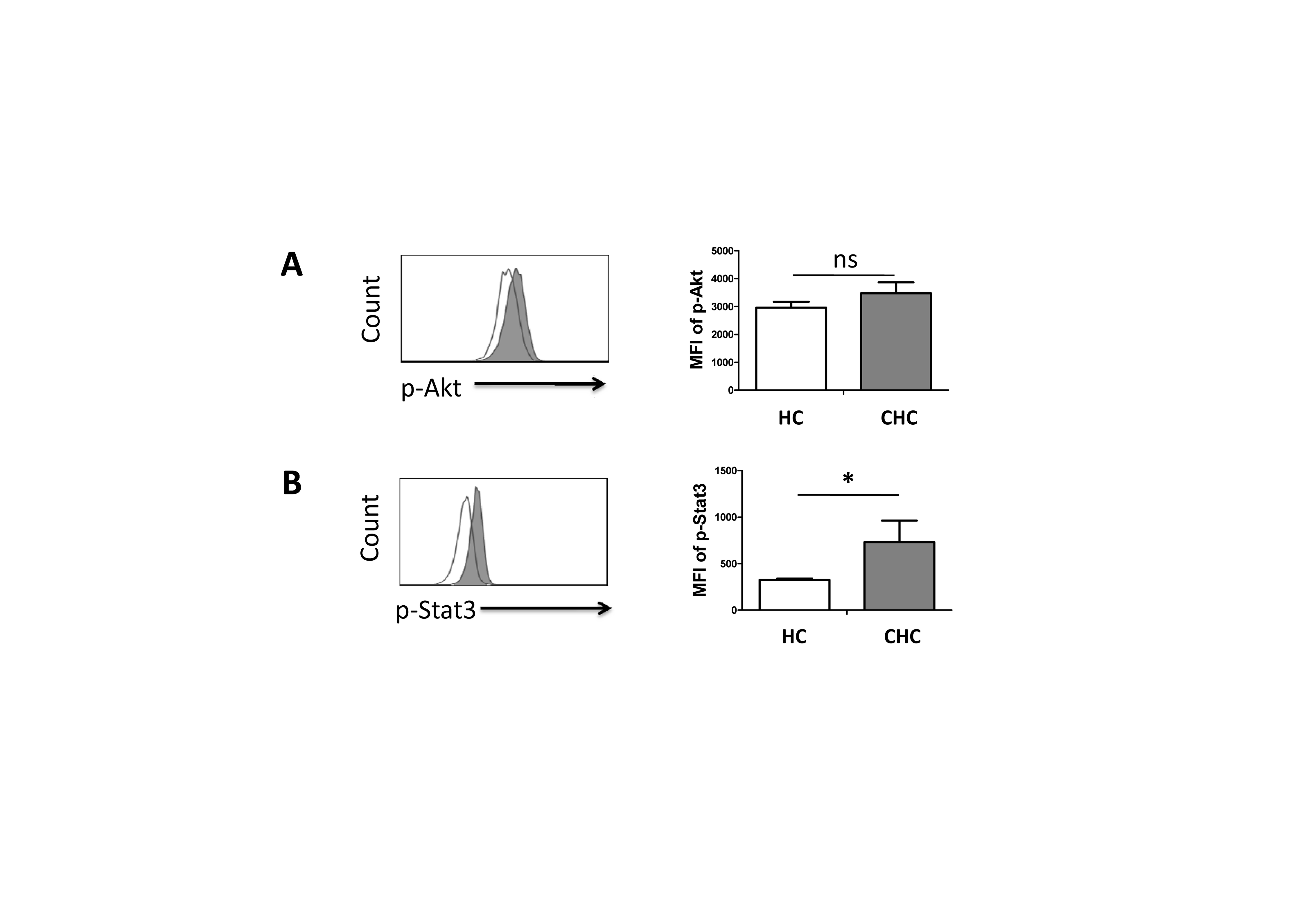

Supplement: S3 Fig — PBMCs were isolated from healthy individuals (unshaded, n = 3) and treatment-naive chronic hepatitis C patients (shaded, n = 5). Cells were stained with anti-CD14-APC-Cy7 antibody, and then fixed for 10 min at 37°C using BD Phosflow™ Fix Buffer I (Cat. No. 557870), permeabilized in BD Phosflow™ Perm Buffer III (Cat. No. 558050) on ice for 30 min. Cells were washed twice and stained with PE-CF594 Mouse Anti-Akt (pS473) antibody and Alexa Fluor 647 Mouse Anti-Stat3 (pY705) antibody for 30 min at room temperature. CD14+ monocytes were analyzed for AKT phosphorylation (A) and STAT3 phosphorylation status (B). Abbreviation: PBMCs, peripheral blood mononuclear cells; HC, healthy control; CHC, chronic hepatitis C patients; MFI, Mean Fluorescence intensity. (TIF) [file pone.0170516.s004.tif]

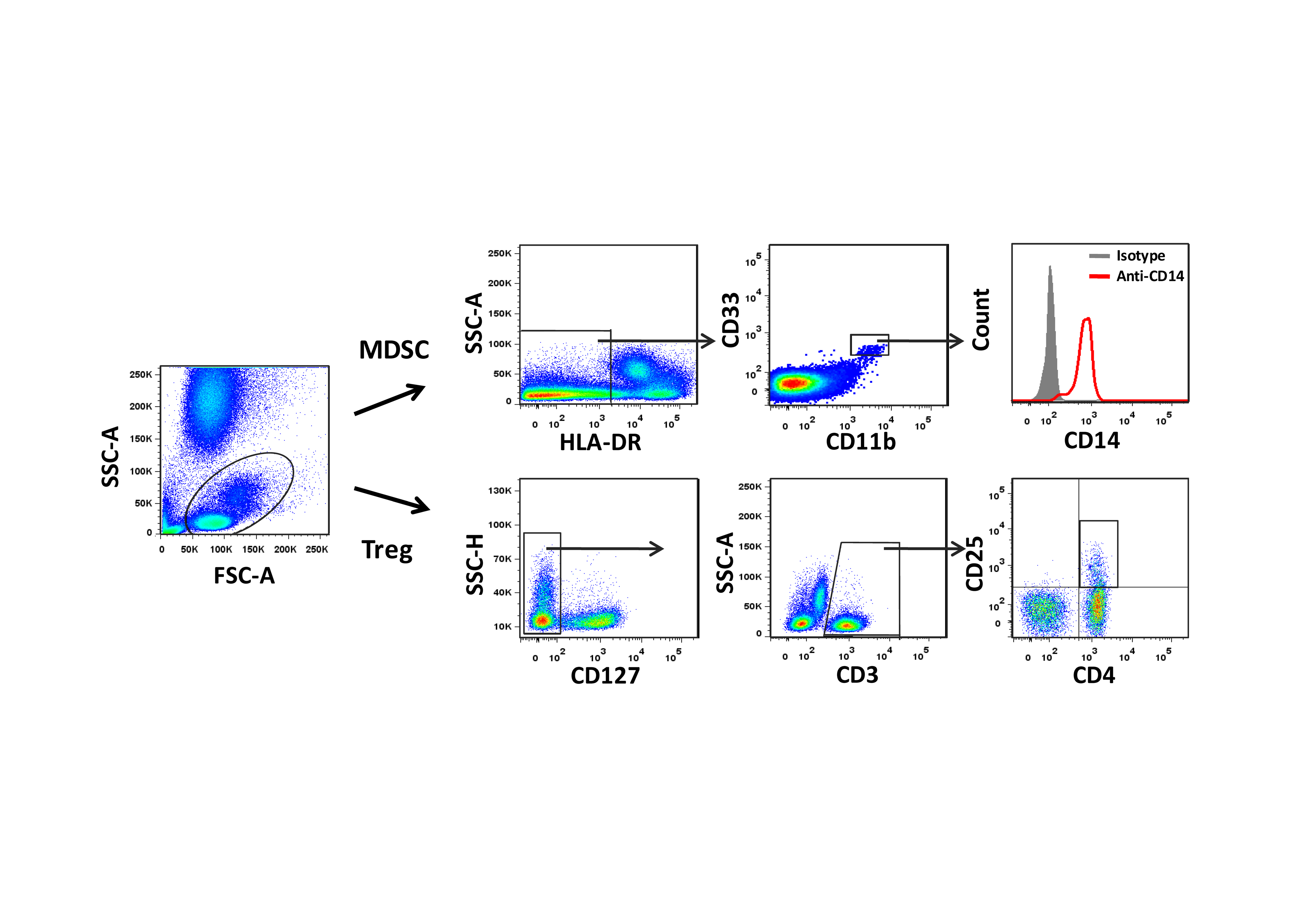

Supplement: S4 Fig — (TIF) [file pone.0170516.s005.tif]

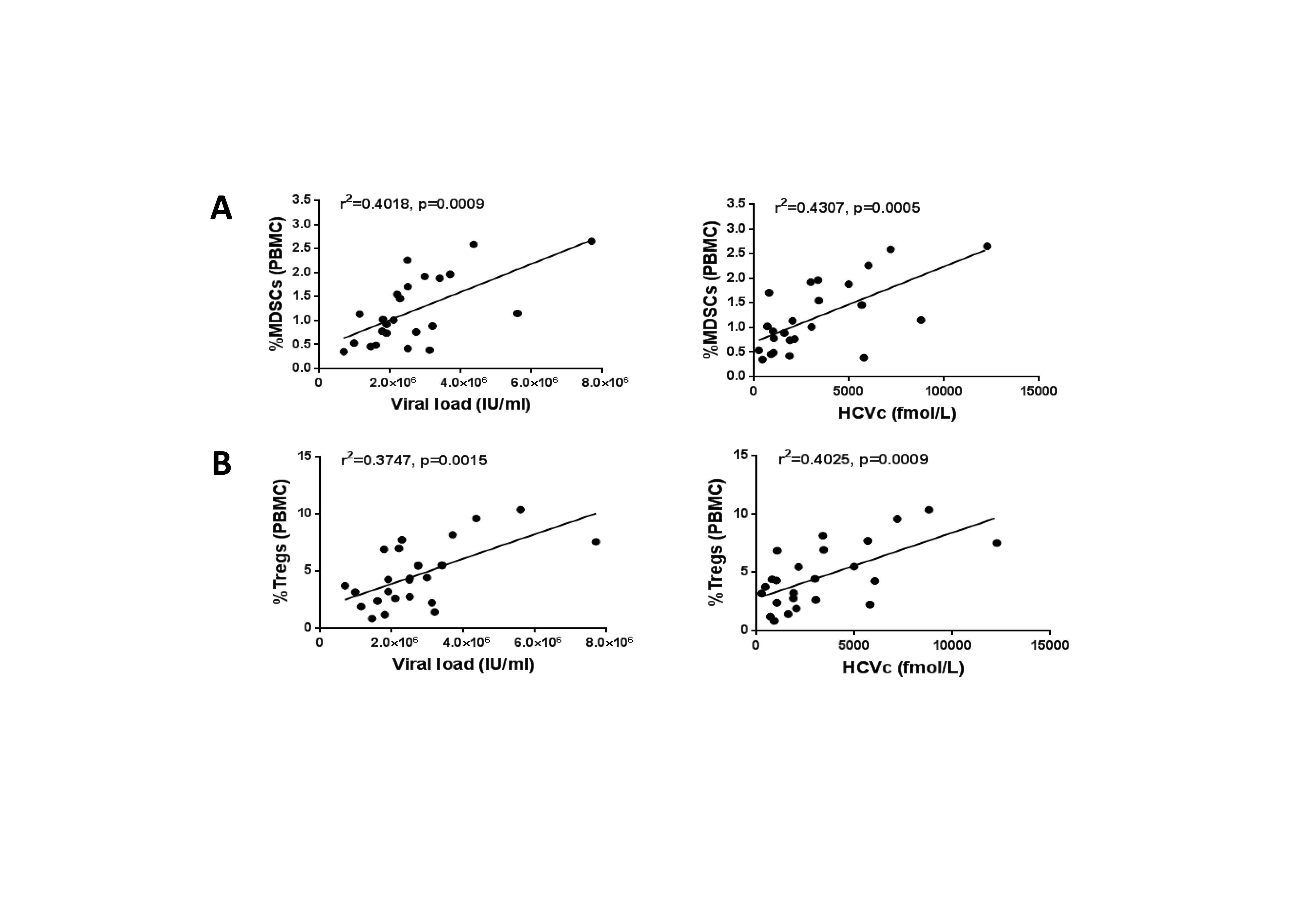

Supplement: S5 Fig — (TIF) [file pone.0170516.s006.tif]

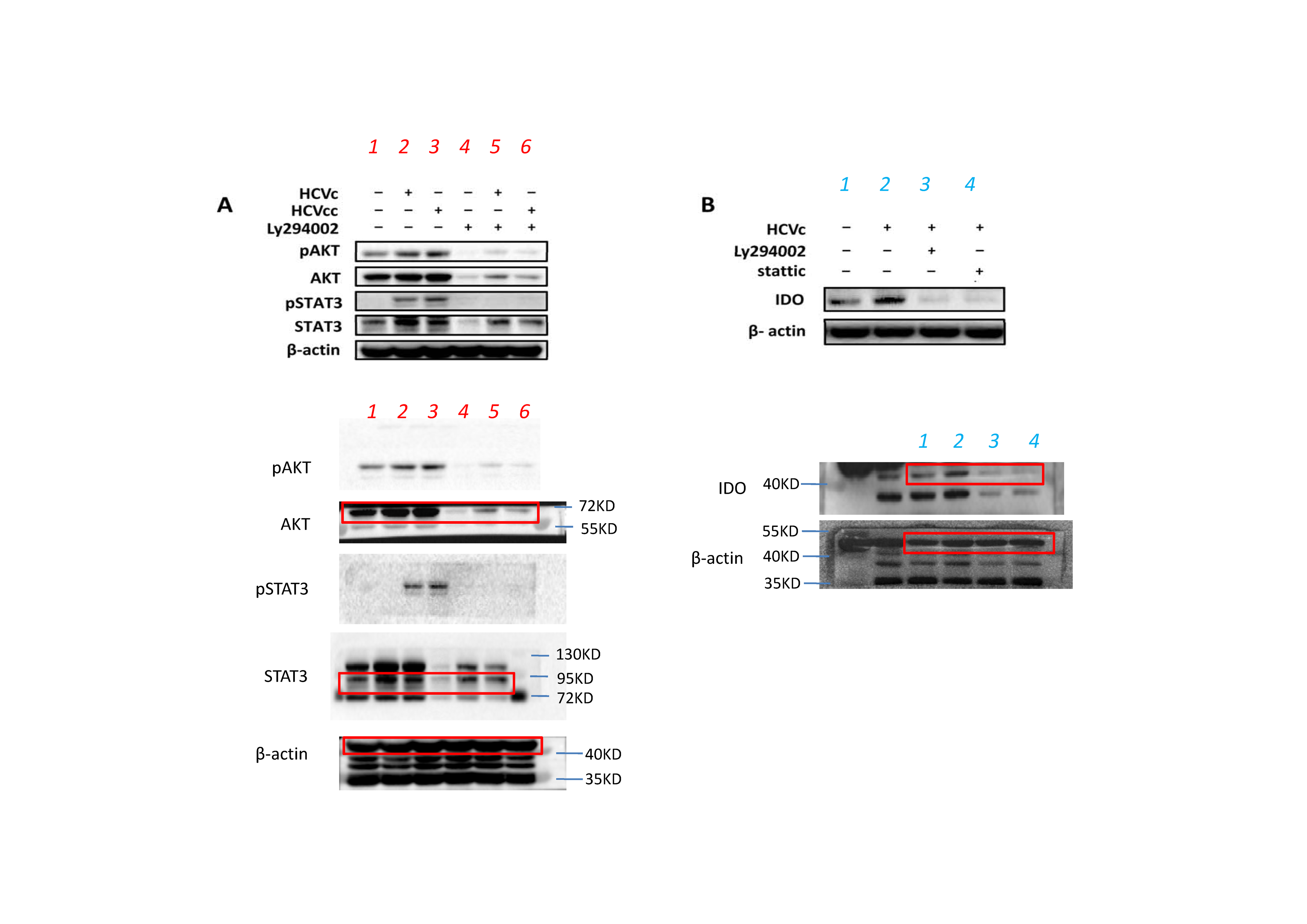

Supplement: S6 Fig — (TIF) [file pone.0170516.s007.tif]
