# Supplementary material for: Hepatitis C Virus Induces MDSCs-Like Monocytes through TLR2/PI3K/AKT/STAT3 Signaling
Source: PLoS One. 2017 Jan 23;12(1):e0170516. doi: 10.1371/journal.pone.0170516 (PMC5256909; doi:10.1371/journal.pone.0170516)
Supplement: S1 Table — (DOCX) [file pone.0170516.s008.docx]

**S1 Table. Characteristics of hepatitis C patients and healthy controls.**

| Clinical data | Chronic HCV infected patients  (n=24) | Healthy controls  (n=16) |
| --- | --- | --- |
| Age, years  (mean±s.e.m.) | 44.9±1.4 | 42.5±1.8 |
| Sex  (Female/male) | 11/13 | 7/9 |
| ALT, U/L  (mean±s.e.m.) | 35.7±4.2 | 32.5±2.5 |
| AST, U/L  (mean±s.e.m.) | 30.2±3.1 | 28.2±1.3 |
| HCV RNA,IU/ml  (mean±s.e.m.) | 2.67×10^6^±0.32×10^6^ | n.a. |

Abbreviations: n.a.: not applicable; SEM: standard error of mean.
