# Supplementary material for: Hepatitis C Virus Induces MDSCs-Like Monocytes through TLR2/PI3K/AKT/STAT3 Signaling
Source: PLoS One. 2017 Jan 23;12(1):e0170516. doi: 10.1371/journal.pone.0170516 (PMC5256909; doi:10.1371/journal.pone.0170516)
Supplement: S2 Table — (DOCX) [file pone.0170516.s009.docx]

**S2 Table. Primer sequences for qRT-PCR.**

| GAPDH, forward | CGGATTTGGTCGTATTGGG |
| --- | --- |
| GAPDH, reverse | TCTCGCTCCTGGAAGATGG |
| IDO1, forward | CAAATCCACGATCATGTGAACC |
| IDO1, reverse | AGAACCCTTCATACACCAGAC |
| IL-10, forward | GGGAGAACCTGAAGACCCTCA |
| IL-10, reverse | TGCTCTTGTTTTCACAGGGAAG |
| HLA-DRα, forward | TGAGGCTCAAGGTGCATTGG |
| HLA-DRα, reverse | GACGTTGGGCTCTCTCAGTT |
| PD-L1, forward | GGACAAGCAGTGACCATCAAG |
| PD-L1, reverse | CCCAGAATTACCAAGTGAGTCCT |
